# The Book World of Medicine and Science

**Published:** 1895-10-26

**Authors:** 


### The Book World of Medicine and Science.

Medical Partnerships, Transfers, and Assistantships, by William Barnard, M.A., LL.B., and G. Bertram Stocker. (London: Stevens and Sons. 1895. Pp. 249. Price 10s. 6d.)

Simple as it may seem to take a partner, to transfer a practice, or to engage an assistant, it is certain that in each of these proceedings there are many pitfalls, and it is impossible to read this very useful handbook without seeing the importance of securing good legal advice when any such matters are in question. Even in the case of those, however, who would never think of moving without the assistance of a lawyer, the volume before us will be found of great assistance whenever such an event has to be discussed; for a consideration of the value of practices, the proper provisions to be introduced into deeds of partnership, and even the most appropriate forms of limitations and restrictions which it is well to enforce when engaging an assistant, are not matters of daily experience either to doctor or lawyer, and a great many hints may be gathered by a careful perusal of the chapters on each subject in this books, together with the notes on the law and the illustrative cases which are given in considerable number. The appendix of cases on restrictive covenants may be particularly referred to, for medical men are far too apt to imagine that they can tie up the practices they dispose of in ways which the Courts very quickly dispose of. The book is well put together, shows a practical acquaintance with the subject of which it treats, and is likely to be very useful.

A Medical and Surgical Help for Shipmasters and Officers in the Merchant Navy. By William Johnson Smith, F.R.C.S., Principal Medical Officer, Seamen's Hospital, Greenwich. (London: Charles Griffin and Co. 1895. Pp. 333.)

This is an admirable book for the particular purpose which it is meant to serve. No doubt it is easy to smile at some of the recommendations contained in it, but the very virtue of the book lies in the fact that its author has endeavoured to describe not necessarily the best imaginable treatment, but such as is within the range of the means and appliances placed at the disposal of ships' officers by the authorised scales of the Board of Trade. It is no small advantage to those for whose guidance it is meant, that it does not recommend the impossible. The book is, indeed, a *multum in parvo.* The hygiene of the ship, ambulance, first aid, nursing, the general anatomy of the body, the uses of the contents of the medicine chest, and the treatment cf a long list of accidents and diseases are dealt with, and finally a chapter is added on cooking on board ship. The directions for the treatment of the various diseases are very good, much emphasis being given to the necessity for careful feeding and nursing. As the author points out, less harm is often likely to result if the attention of unqualified persons is largely given to the warding off of further evil and promoting the comfort and physical strength of the patient, than if active interference is undertaken by means of surgical instruments and powerful drugs. In the surgical portion full recognition is given to the necessity of using such antiseptics as are available, in relation to which it is interesting to find the author strongly recommending that, when the weather will allow, severe surgical injuries should be treated in a tent rigged upon deck, or in a small boat on deck, rather than in the too often foul forecastle. The book is interesting, reliable, and in the ambulance portion is very well illustrated. It certainly should find a place not only in the officers' cabin but in the forecastle; for rough as sailors often are, there are some in almost every crew who would be thoroughly interested in much that this book contains, and might by its perusal become of much help in many serious emergencies.

The Case against Butchers' Meat. By Charles Forward. (Manchester: The Vegetarian Society. Price 1s.)

As the title plainly states that this is the case against butchers' meat, we must take it for what it is worth, and must not quarrel with its author for presenting a merely one-sided statement of the question. For general readers the complete absence of the judicial spirit is a disadvantage, for however honest an advocate may be, we may be sure that truth does not lie all on one side. Still, if this brochure is read on the understanding that it represents the very utmost that can be said against the general verdict of the human race in favour of a mixed diet, it will not be entirely devoid of usefulness.

But we would remark that the advocate must accept responsibility for his weak, as well as his strong arguments, and that all the economic dogmas which are here put forward in regard to the land and the unemployed and the relation of wages to the cost of living, only support the vegetarian creed so long as they themselves turn out to be true, which is far from proven yet.

In regard to the physiological, humane, and moral arguments adduced, while admitting that they are stated in a striking way, we can by no means admit that they express the whole truth. Everyone knows that great evils and abuses exist in the meat trade, and that cruelty is rampant in every step of man's dealings with animals. All these abuses we would gladly see reformed, but to throw man back upon vegetarian diet is another affair, for the necessity of which no proof has yet been adduced—certainly not in this book.

Memories of Seven Campaigns. By James Howard Thornton, C.B., M.B. (London: Constable and Co. 1895).

Dr. James Thornton's memoirs of the seven campaigns in which he served have just been published and presented to the public in illustrated one-volume form. Besides embracing a wide historical and geographical area, the book is most interesting as a record of 35 years' service in the medical department in India, China, and the Soudan. Dr. Thornton began his career as a military surgeon at a critical period in the modern history of our Empire. His work was still fresh to him when the news spread through India that the native army was in open revolt. The two years which followed, and during which the mutiny lasted were years of severe apprenticeship to him, and it is round this period where, perhaps, the greatest historical interest of the volume centres. Besides his campaigns the writer had other than actual human foes to combat with; the ills the flesh is heir to in tropical climates were constant assailants; and the pages are full of the matured reflections of an intelligent physician's mind on questions of preventive medicines. We have heard it elsewhere, but it is gratifying to see it reiterated again on Dr. Thornton's own personal authority, that sanitary improvements in the supply of pure water in India have done much to keep down the ravages of cholera. There have been many significant alterations in the Medical Department during the period our author served in it, but it is open to many improvements still. Dr. Thornton's book, which is written with a certain force, and a perspicuous directness of language, is prefaced by A. Egmont Hake, author of "Chinese Gordon," &c.

